# (3*S*,7*R*)-7,14,16-Trihy­droxy-3-methyl-3,4,5,6,7,8,9,10,11,12-deca­hydro-1*H*-2-benzoxacyclo­tetra­decin-1-one.

**DOI:** 10.1107/S1600536812041141

**Published:** 2012-10-06

**Authors:** Sarah Drzymala, Werner Kraus, Franziska Emmerling, Matthias Koch

**Affiliations:** a BAM Federal Institute for Materials Research and Testing, Department of Analytical Chemistry, Reference Materials, Richard-Willstätter-Strasse 11, D-12489 Berlin, Germany

## Abstract

The asymmetric unit of the title compound, C_18_H_26_O_5_, which is known as α-zearalanol, contains two mol­ecules having the same conformation, with a r.m.s. deviation of less than 0.03 Å for all non-H atoms. In each independent mol­ecule, an intra­molecular O—H⋯O hydrogen bond stabilizes the mol­ecular conformation. In the crystal, O—H⋯O hydrogen bonds link the mol­ecules, forming infinite chains along [110] and [1-10].

## Related literature

For the chemical preparation of α-zearalanol, see: Urry *et al.* (1966 ▶). For its natural occurrence as a metabolite, see: Baldwin *et al.* (1983 ▶) and for its use as an animal growth promoter, see: Wang & Wang (2007 ▶). For the crystal structures of related derivatives, see: Panneerselvam *et al.* (1996 ▶); Gelo-Pujić *et al.* (1994 ▶); Zhao *et al.* (2008 ▶); Köppen *et al.* (2012 ▶); Drzymala *et al.* (2012 ▶)*.* 
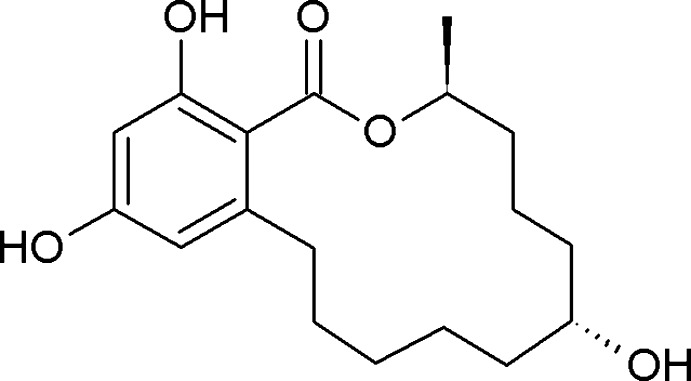


## Experimental

### 

#### Crystal data

C_18_H_26_O_5_
*M* *_r_* = 322.39
Triclinic, 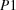

*a* = 5.0734 (11) Å
*b* = 11.618 (2) Å
*c* = 14.718 (3) Å
α = 87.388 (13)°
β = 86.595 (15)°
γ = 89.780 (15)°
*V* = 865.0 (3) Å^3^
*Z* = 2
Mo *K*α radiation
μ = 0.09 mm^−1^
*T* = 296 K
0.43 × 0.22 × 0.10 mm

#### Data collection

Bruker APEXII CCD diffractometer
Absorption correction: multi-scan (*SADABS*; Bruker, 2001 ▶) *T*_min_ = 0.186, *T*_max_ = 0.350
19642 measured reflections
4264 independent reflections
3421 reflections with *I* > 2σ(*I*)
*R*_int_ = 0.095

#### Refinement

*R*[*F* ^2^ > 2σ(*F* ^2^)] = 0.059
*wR*(*F* ^2^) = 0.147
*S* = 0.95
4264 reflections
431 parameters
7 restraints
H atoms treated by a mixture of independent and constrained refinement
Δρ_max_ = 0.24 e Å^−3^
Δρ_min_ = −0.16 e Å^−3^

### 

Data collection: *APEX2* (Bruker, 2001 ▶); cell refinement: *SAINT* (Bruker, 2001 ▶); data reduction: *SAINT*; program(s) used to solve structure: *SHELXS97* (Sheldrick, 2008 ▶); program(s) used to refine structure: *SHELXL97* (Sheldrick, 2008 ▶); molecular graphics: *SHELXTL* (Sheldrick, 2008 ▶); software used to prepare material for publication: *SHELXTL*.

## Supplementary Material

Crystal structure: contains datablock(s) I, New_Global_Publ_Block. DOI: 10.1107/S1600536812041141/fj2595sup1.cif

Structure factors: contains datablock(s) I. DOI: 10.1107/S1600536812041141/fj2595Isup2.hkl

Additional supplementary materials:  crystallographic information; 3D view; checkCIF report

## Figures and Tables

**Table 1:** Hydrogen-bond geometry (Å, °)

*D*—H⋯*A*	*D*—H	H⋯*A*	*D*⋯*A*	*D*—H⋯*A*
O5—H5*A*⋯O2	0.82	1.83	2.549 (3)	146
O5′—H5′*A*⋯O2′	0.82	1.82	2.540 (3)	146
O4—H4*A*⋯O3^i^	0.83 (2)	1.93 (3)	2.745 (3)	171 (3)
O4′—H4′*A*⋯O3′^ii^	0.82 (3)	1.94 (3)	2.740 (3)	163 (4)
O3—H3*A*⋯O4^iii^	0.82 (3)	2.29 (3)	3.080 (3)	162 (3)
O3′—H3′*A*⋯O4′^iii^	0.82 (3)	2.27 (3)	3.067 (3)	165 (4)

Symmetry codes: (i) 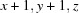; (ii) 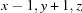; (iii) 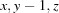.

**Fig. 1.**
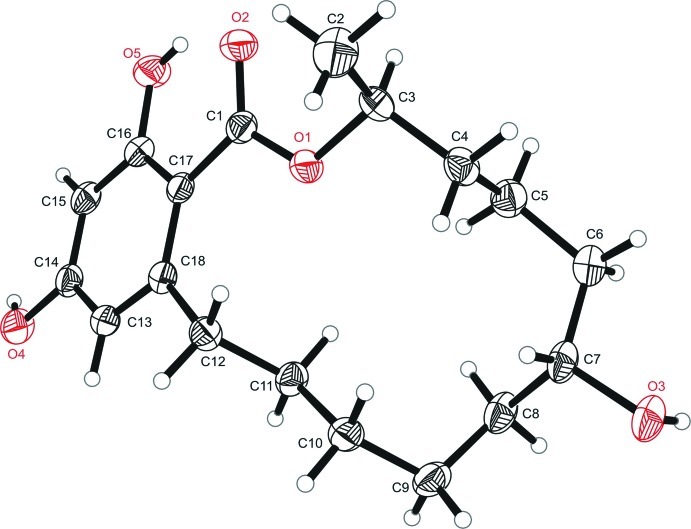
: ORTEP representation of the title compound with atomic labeling shown with 30% probability displacement ellipsoids.

**Fig. 2.**
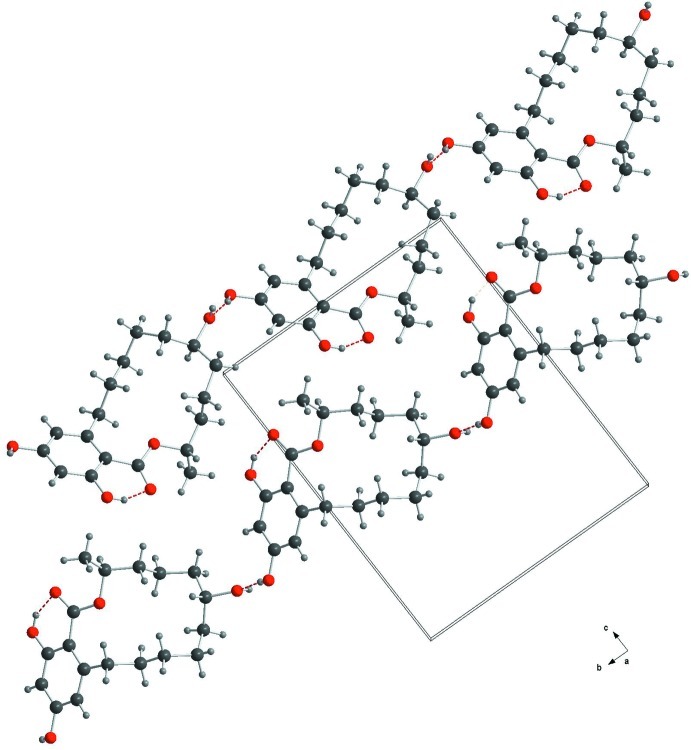
: View of the unit cell of the title compound, showing the hydrogen-bonded chains of the two independent molecules. Hydrogen bonds are drawn as dashed red lines.

**Fig. 3.**
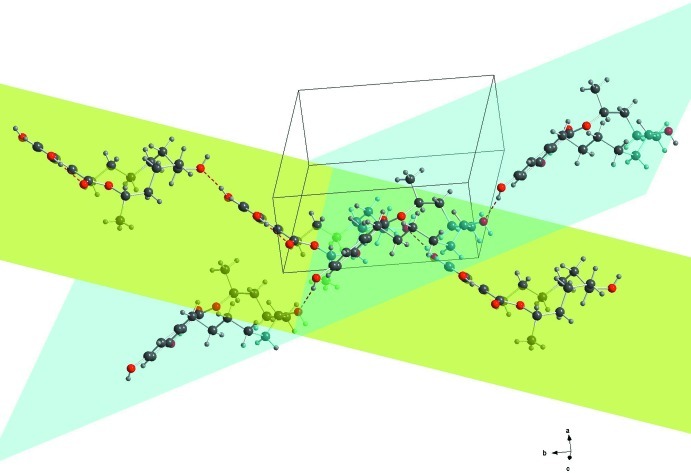
: View of the unit cell of the title compound, showing the two chains with planes of the basevectors. Turquoise for [1 1 0] and lime for [1 - 1 0]. Hydrogen bonds are drawn as dashed red lines.

**Fig. 4.**
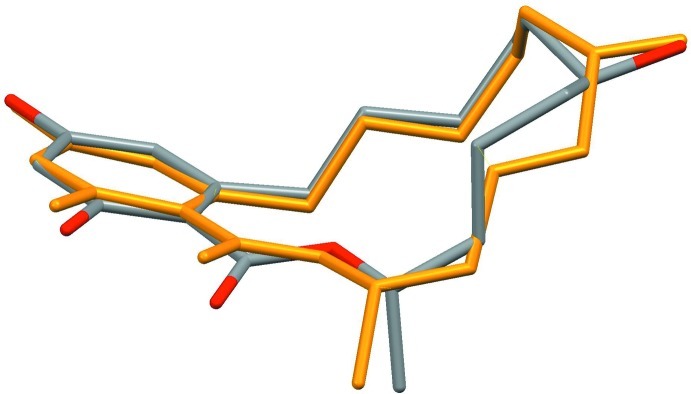
: The difference in conformation between the known β-Zearalanol (yellow, Gelo-Pujić et al., 1994) and the title compound.

## supplementary crystallographic information

### Comment

α-Zearalanol (α-ZAL, generic name Zeranol) is a resorcylic acid lactone (RAL) with estrogenic and anabolic activity. α-ZAL can be obtained chemically by reduction of zearalenone (ZEN) (Urry *et al.* 1966), a mycotoxin produced by a variety of *Fusarium* fungi and well known crop contaminant. α-ZAL also occurs naturally as a metabolite of zearalanone (ZAN), another ZEN derivative (Baldwin *et al.*, 1983). Crystal structures of ZEN and ZEN derivatives have been elucidated by Panneerselvam *et al.* (1996), Gelo-Pujić *et al.* (1994), Zhao *et al.* (2008), Köppen *et al.* (2012) and Drzymala *et al.* (2012)*.*

ZEN-related structures have a more or less pronounced hormonal activity. Particularly α-ZAL proved to be an effective anabolic hormone. Marketed under the trade name Ralgro, it is widely used as a growth promoter in cattle. In contrast to the U.S.A., Canada and several other countries, α-Zearalanol was banned by the EU in 1985 (Wang & Wang, 2007) resulting in a series of legal issues between the US and the EU. Due to its growth promoting effects α-ZAL also belongs to the list of substances prohibited in sports as classified by the World Anti-Doping Agency.

The compound has a macrocyclic structure and crystallizes in the triclinic space group *P*1. The molecular structure of the compound and the atom-labeling scheme are shown in Fig 1. The absolute configuration could not be defined confidently based on the single-crystal diffraction data. The isomeric purity of the title compound was confirmed by ^1^H-NMR, HPLC-DAD and –MS/MS data. Fig. 4 shows the difference in conformation between the known β-Zearalanol (Gelo-Pujić *et al.*, 1994) and the title compound. Every molecule in the asymmetric unit builds an infinite chain with the help of hydrogen bonds of the hydroxyl groups. The two chains in relation to the unit cell are depicted in Fig. 2. The analysis of polymeric structures shows two infinite one dimensional chains with the base vectors of [1 1 0] and [1 - 1 0], Fig 3.

### Experimental

α-Zearalanol was obtained from Sigma-Aldrich Chemie GmbH (Germany, purity 97.0%). 5 mg (15.5 *µ*mol) were weighed in a 1.5 ml HPLC glass vial and solved in 0.6 ml diethyl ether. Subsequently, 0.2 ml of *n*-hexane were added. Colorless crystals of the title compound were formed after 14 days of slow solvent evaporation at room temperature.

### Refinement

All H-atoms were positioned geometrically and refined using a riding model with d(C—H) = 0.93 Å, *U*_iso_=1.2*U*_eq_ (C) for aromatic 0.98 Å, *U*_iso_ = 1.2*U*_eq_ (C) for CH, 0.97 Å, *U*_iso_ = 1.2*U*_eq_ (C) for CH_2_, 0.96 Å, *U*_iso_ = 1.5*U*_eq_ (C) for CH_3_ atoms, and 0.82 Å, *U*_iso_ = 1.5*U*_eq_ (C) for hydroxyl group of O5. The hydrogen atoms from the other hydroxyl groups were treated independently. In the absence of significant anomalous dispersion effects 3785 Friedel pairs were merged. The absolute configuration has not been determined by anomalous-dispersion effects in diffraction measurements of the crystal. The enantiomer has been assigned by reference to an unchanging chiral centre in the synthetic procedure.

### Crystal data

**Table:**

C_18_H_26_O_5_	*Z* = 2
*M**_r_* = 322.39	*F*(000) = 348
Triclinic, *P*1	*D*_x_ = 1.238 Mg m^−^^3^
*a* = 5.0734 (11) Å	Mo *K*α radiation, λ = 0.71073 Å
*b* = 11.618 (2) Å	Cell parameters from 6563 reflections
*c* = 14.718 (3) Å	θ = 2.3–26.4°
α = 87.388 (13)°	µ = 0.09 mm^−^^1^
β = 86.595 (15)°	*T* = 296 K
γ = 89.780 (15)°	Block, colourless
*V* = 865.0 (3) Å^3^	0.43 × 0.22 × 0.10 mm

### Data collection

**Table:**

Bruker APEXII CCD diffractometer	4264 independent reflections
Radiation source: fine-focus sealed tube	3421 reflections with *I* > 2σ(*I*)
Graphite monochromator	*R*_int_ = 0.095
φ and ω scans	θ_max_ = 28.3°, θ_min_ = 1.4°
Absorption correction: multi-scan (*SADABS*; Bruker, 2001)	*h* = −6→6
*T*_min_ = 0.186, *T*_max_ = 0.350	*k* = −15→15
19642 measured reflections	*l* = −19→19

### Refinement

**Table:**

Refinement on *F*^2^	Primary atom site location: structure-invariant direct methods
Least-squares matrix: full	Secondary atom site location: difference Fourier map
*R*[*F*^2^ > 2σ(*F*^2^)] = 0.059	Hydrogen site location: inferred from neighbouring sites
*wR*(*F*^2^) = 0.147	H atoms treated by a mixture of independent and constrained refinement
*S* = 0.95	*w* = 1/[σ^2^(*F*_o_^2^) + (0.0803*P*)^2^] where *P* = (*F*_o_^2^ + 2*F*_c_^2^)/3
4264 reflections	(Δ/σ)_max_ < 0.001
431 parameters	Δρ_max_ = 0.24 e Å^−^^3^
7 restraints	Δρ_min_ = −0.16 e Å^−^^3^

### Special details

**Table:**

Geometry. All e.s.d.'s (except the e.s.d. in the dihedral angle between two l.s. planes) are estimated using the full covariance matrix. The cell e.s.d.'s are taken into account individually in the estimation of e.s.d.'s in distances, angles and torsion angles; correlations between e.s.d.'s in cell parameters are only used when they are defined by crystal symmetry. An approximate (isotropic) treatment of cell e.s.d.'s is used for estimating e.s.d.'s involving l.s. planes.
Refinement. Refinement of *F*^2^ against ALL reflections. The weighted *R*-factor *wR* and goodness of fit *S* are based on *F*^2^, conventional *R*-factors *R* are based on *F*, with *F* set to zero for negative *F*^2^. The threshold expression of *F*^2^ > σ(*F*^2^) is used only for calculating *R*-factors(gt) *etc*. and is not relevant to the choice of reflections for refinement. *R*-factors based on *F*^2^ are statistically about twice as large as those based on *F*, and *R*- factors based on ALL data will be even larger.

### Fractional atomic coordinates and isotropic or equivalent isotropic displacement parameters (Å^2^)

**Table:**

	*x*	*y*	*z*	*U*_iso_*/*U*_eq_	
O1	−0.1448 (4)	0.89233 (14)	0.65863 (11)	0.0465 (4)	
O2	0.0413 (4)	0.99572 (16)	0.76207 (12)	0.0555 (5)	
O3	−0.3004 (5)	0.45828 (17)	0.45181 (16)	0.0618 (6)	
O4	0.2440 (5)	1.34395 (16)	0.41989 (13)	0.0570 (5)	
O5	0.3693 (4)	1.15285 (17)	0.70652 (13)	0.0578 (5)	
H5A	0.3015	1.1038	0.7423	0.087*	
C1	−0.0231 (5)	0.9872 (2)	0.68358 (16)	0.0409 (5)	
C2	−0.4309 (7)	0.8317 (3)	0.7915 (2)	0.0683 (8)	
H2A	−0.3901	0.9000	0.8222	0.102*	
H2B	−0.4694	0.7698	0.8356	0.102*	
H2C	−0.5817	0.8459	0.7561	0.102*	
C3	−0.1985 (5)	0.7995 (2)	0.72962 (16)	0.0435 (6)	
H3B	−0.0429	0.7882	0.7654	0.052*	
C4	−0.2456 (5)	0.6918 (2)	0.67829 (18)	0.0469 (6)	
H4B	−0.3192	0.6329	0.7211	0.056*	
H4C	−0.3763	0.7093	0.6341	0.056*	
C5	−0.0035 (6)	0.6437 (2)	0.6292 (2)	0.0526 (7)	
H5B	0.1227	0.6215	0.6738	0.063*	
H5C	0.0767	0.7042	0.5893	0.063*	
C6	−0.0549 (7)	0.5390 (2)	0.5722 (2)	0.0580 (7)	
H6A	0.1135	0.5081	0.5500	0.070*	
H6B	−0.1424	0.4797	0.6115	0.070*	
C7	−0.2229 (6)	0.5653 (2)	0.49103 (19)	0.0493 (6)	
H7A	−0.3846	0.6033	0.5140	0.059*	
C8	−0.0915 (6)	0.6456 (2)	0.41628 (19)	0.0535 (7)	
H8A	0.0351	0.6937	0.4435	0.064*	
H8B	0.0053	0.5991	0.3725	0.064*	
C9	−0.2828 (6)	0.7229 (2)	0.36581 (18)	0.0563 (7)	
H9A	−0.1933	0.7532	0.3097	0.068*	
H9B	−0.4294	0.6763	0.3493	0.068*	
C10	−0.3925 (6)	0.8235 (2)	0.41891 (19)	0.0482 (6)	
H10A	−0.4831	0.7935	0.4749	0.058*	
H10B	−0.5218	0.8637	0.3833	0.058*	
C11	−0.1827 (5)	0.9100 (2)	0.44291 (17)	0.0444 (6)	
H11A	−0.0530	0.8701	0.4787	0.053*	
H11B	−0.0925	0.9408	0.3871	0.053*	
C12	−0.2995 (5)	1.0093 (2)	0.49636 (17)	0.0411 (5)	
H12A	−0.4017	0.9785	0.5497	0.049*	
H12B	−0.4182	1.0532	0.4587	0.049*	
C13	−0.0089 (5)	1.1798 (2)	0.46580 (17)	0.0438 (6)	
H13A	−0.0918	1.1891	0.4113	0.053*	
C14	0.1884 (5)	1.2568 (2)	0.48410 (17)	0.0437 (6)	
C15	0.3129 (6)	1.2462 (2)	0.56527 (18)	0.0457 (6)	
H15A	0.4448	1.2977	0.5775	0.055*	
C16	0.2384 (5)	1.1575 (2)	0.62849 (16)	0.0415 (5)	
C17	0.0383 (5)	1.07732 (19)	0.61033 (16)	0.0372 (5)	
C18	−0.0857 (5)	1.08946 (19)	0.52648 (16)	0.0377 (5)	
O1'	0.5442 (4)	0.37345 (14)	0.94217 (11)	0.0462 (4)	
O2'	0.3680 (4)	0.48975 (16)	0.83705 (12)	0.0549 (5)	
O3'	0.6823 (5)	−0.08133 (17)	1.15210 (15)	0.0602 (5)	
O4'	0.1425 (4)	0.79967 (16)	1.17536 (14)	0.0567 (5)	
O5'	0.0323 (4)	0.63890 (17)	0.89001 (13)	0.0608 (5)	
H5'A	0.1034	0.5941	0.8547	0.091*	
C1'	0.4249 (5)	0.4708 (2)	0.91655 (17)	0.0414 (5)	
C2'	0.8444 (7)	0.3297 (3)	0.8122 (3)	0.0726 (9)	
H2'A	0.8052	0.4014	0.7807	0.109*	
H2'B	0.8904	0.2729	0.7686	0.109*	
H2'C	0.9897	0.3403	0.8498	0.109*	
C3'	0.6061 (5)	0.2899 (2)	0.87071 (17)	0.0436 (6)	
H3'B	0.4548	0.2832	0.8329	0.052*	
C4'	0.6511 (6)	0.1755 (2)	0.92215 (19)	0.0484 (6)	
H4'B	0.7733	0.1878	0.9690	0.058*	
H4'C	0.7338	0.1224	0.8801	0.058*	
C5'	0.4010 (6)	0.1196 (2)	0.96673 (18)	0.0497 (6)	
H5'B	0.2853	0.1009	0.9194	0.060*	
H5'C	0.3099	0.1750	1.0047	0.060*	
C6'	0.4513 (6)	0.0096 (2)	1.02532 (19)	0.0548 (7)	
H6'A	0.2827	−0.0263	1.0432	0.066*	
H6'B	0.5524	−0.0437	0.9882	0.066*	
C7'	0.5984 (6)	0.0283 (2)	1.11146 (18)	0.0472 (6)	
H7'A	0.7575	0.0733	1.0933	0.057*	
C8'	0.4374 (6)	0.0953 (2)	1.18242 (19)	0.0511 (6)	
H8'A	0.3308	0.0415	1.2210	0.061*	
H8'B	0.3183	0.1471	1.1514	0.061*	
C9'	0.6079 (7)	0.1660 (2)	1.24287 (19)	0.0594 (8)	
H9'A	0.4982	0.1905	1.2947	0.071*	
H9'B	0.7455	0.1166	1.2660	0.071*	
C10'	0.7366 (6)	0.2724 (2)	1.1937 (2)	0.0537 (7)	
H10C	0.8532	0.3073	1.2345	0.064*	
H10D	0.8440	0.2479	1.1414	0.064*	
C11'	0.5409 (6)	0.3635 (2)	1.16147 (18)	0.0491 (6)	
H11C	0.4388	0.3909	1.2140	0.059*	
H11D	0.4194	0.3281	1.1227	0.059*	
C12'	0.6742 (5)	0.4672 (2)	1.10867 (18)	0.0440 (6)	
H12C	0.7903	0.5050	1.1480	0.053*	
H12D	0.7810	0.4401	1.0571	0.053*	
C13'	0.3928 (6)	0.6373 (2)	1.13423 (18)	0.0456 (6)	
H13B	0.4704	0.6401	1.1898	0.055*	
C14'	0.1995 (5)	0.7179 (2)	1.11290 (17)	0.0444 (6)	
C15'	0.0797 (6)	0.7162 (2)	1.03129 (18)	0.0450 (6)	
H15B	−0.0523	0.7690	1.0180	0.054*	
C16'	0.1590 (5)	0.6347 (2)	0.96934 (16)	0.0417 (6)	
C17'	0.3551 (5)	0.55111 (19)	0.98913 (16)	0.0376 (5)	
C18'	0.4720 (5)	0.55347 (19)	1.07492 (16)	0.0389 (5)	
H4A	0.388 (4)	1.371 (3)	0.431 (2)	0.073 (11)*	
H3'A	0.543 (5)	−0.113 (4)	1.169 (3)	0.108 (17)*	
H3A	−0.175 (5)	0.418 (3)	0.436 (3)	0.087 (14)*	
H4'A	−0.005 (5)	0.823 (4)	1.163 (4)	0.13 (2)*	

### Atomic displacement parameters (Å^2^)

**Table:**

	*U*^11^	*U*^22^	*U*^33^	*U*^12^	*U*^13^	*U*^23^
O1	0.0625 (12)	0.0364 (9)	0.0408 (9)	−0.0108 (8)	−0.0077 (8)	0.0041 (7)
O2	0.0760 (13)	0.0506 (10)	0.0408 (9)	−0.0087 (10)	−0.0120 (9)	−0.0022 (8)
O3	0.0717 (16)	0.0379 (11)	0.0762 (14)	−0.0139 (11)	−0.0031 (12)	−0.0090 (9)
O4	0.0772 (15)	0.0375 (10)	0.0557 (11)	−0.0172 (10)	−0.0011 (11)	0.0022 (8)
O5	0.0702 (13)	0.0535 (11)	0.0519 (11)	−0.0147 (10)	−0.0194 (10)	−0.0050 (9)
C1	0.0458 (14)	0.0372 (12)	0.0393 (13)	0.0009 (10)	0.0025 (10)	−0.0048 (9)
C2	0.066 (2)	0.0638 (19)	0.072 (2)	−0.0032 (16)	0.0199 (16)	−0.0031 (15)
C3	0.0473 (14)	0.0422 (13)	0.0400 (12)	−0.0025 (11)	−0.0010 (10)	0.0090 (10)
C4	0.0490 (15)	0.0413 (14)	0.0494 (14)	−0.0106 (11)	−0.0020 (11)	0.0078 (11)
C5	0.0513 (16)	0.0489 (15)	0.0584 (16)	−0.0009 (13)	−0.0098 (13)	−0.0034 (12)
C6	0.072 (2)	0.0400 (14)	0.0628 (17)	−0.0007 (13)	−0.0070 (15)	−0.0040 (12)
C7	0.0560 (16)	0.0319 (12)	0.0593 (16)	−0.0116 (11)	0.0059 (13)	−0.0059 (11)
C8	0.0623 (18)	0.0402 (14)	0.0568 (16)	−0.0132 (13)	0.0130 (14)	−0.0098 (12)
C9	0.078 (2)	0.0482 (15)	0.0433 (13)	−0.0210 (14)	−0.0054 (13)	−0.0057 (11)
C10	0.0565 (16)	0.0425 (13)	0.0466 (13)	−0.0088 (12)	−0.0128 (12)	0.0004 (10)
C11	0.0509 (15)	0.0368 (12)	0.0456 (13)	−0.0098 (11)	−0.0031 (11)	−0.0029 (10)
C12	0.0397 (13)	0.0377 (12)	0.0462 (13)	−0.0041 (10)	−0.0068 (10)	0.0001 (10)
C13	0.0559 (16)	0.0344 (12)	0.0420 (13)	−0.0015 (11)	−0.0082 (11)	−0.0033 (10)
C14	0.0554 (15)	0.0291 (11)	0.0461 (13)	−0.0034 (11)	0.0019 (11)	−0.0035 (10)
C15	0.0515 (14)	0.0356 (12)	0.0506 (14)	−0.0083 (11)	−0.0005 (11)	−0.0105 (10)
C16	0.0462 (14)	0.0379 (13)	0.0413 (13)	−0.0012 (11)	−0.0031 (11)	−0.0091 (10)
C17	0.0438 (13)	0.0297 (11)	0.0384 (11)	−0.0005 (10)	−0.0001 (10)	−0.0067 (9)
C18	0.0377 (12)	0.0324 (11)	0.0432 (12)	0.0003 (10)	−0.0024 (10)	−0.0050 (9)
O1'	0.0606 (11)	0.0368 (9)	0.0418 (9)	0.0061 (8)	−0.0066 (8)	−0.0057 (7)
O2'	0.0787 (14)	0.0458 (10)	0.0406 (10)	0.0043 (9)	−0.0097 (9)	0.0003 (8)
O3'	0.0700 (15)	0.0376 (10)	0.0724 (13)	0.0094 (10)	−0.0038 (11)	0.0034 (9)
O4'	0.0712 (14)	0.0415 (11)	0.0567 (11)	0.0081 (10)	0.0065 (10)	−0.0081 (8)
O5'	0.0785 (14)	0.0532 (11)	0.0521 (11)	0.0131 (10)	−0.0191 (10)	0.0004 (9)
C1'	0.0474 (14)	0.0343 (12)	0.0420 (13)	−0.0040 (10)	−0.0006 (11)	0.0016 (9)
C2'	0.070 (2)	0.0622 (19)	0.082 (2)	−0.0024 (17)	0.0238 (18)	−0.0024 (16)
C3'	0.0473 (14)	0.0415 (13)	0.0417 (12)	−0.0006 (11)	0.0031 (10)	−0.0074 (10)
C4'	0.0502 (15)	0.0445 (14)	0.0504 (14)	0.0071 (12)	0.0012 (11)	−0.0080 (11)
C5'	0.0538 (16)	0.0470 (15)	0.0486 (14)	−0.0032 (12)	−0.0060 (12)	−0.0012 (11)
C6'	0.0695 (19)	0.0388 (14)	0.0564 (16)	−0.0056 (13)	−0.0047 (14)	−0.0040 (12)
C7'	0.0563 (16)	0.0320 (12)	0.0528 (14)	0.0023 (11)	0.0000 (12)	−0.0001 (10)
C8'	0.0619 (17)	0.0403 (13)	0.0499 (14)	0.0036 (12)	0.0029 (12)	0.0013 (11)
C9'	0.093 (2)	0.0404 (14)	0.0452 (14)	0.0140 (15)	−0.0131 (15)	0.0002 (11)
C10'	0.0653 (18)	0.0387 (13)	0.0596 (17)	0.0054 (12)	−0.0225 (14)	−0.0040 (12)
C11'	0.0591 (16)	0.0382 (13)	0.0500 (14)	0.0053 (12)	−0.0073 (12)	0.0019 (11)
C12'	0.0495 (15)	0.0388 (13)	0.0452 (13)	0.0012 (11)	−0.0107 (11)	−0.0059 (10)
C13'	0.0550 (16)	0.0367 (13)	0.0452 (13)	−0.0047 (11)	−0.0045 (11)	−0.0019 (10)
C14'	0.0549 (15)	0.0313 (12)	0.0452 (13)	−0.0010 (11)	0.0090 (11)	0.0015 (10)
C15'	0.0498 (15)	0.0321 (12)	0.0521 (14)	0.0041 (11)	0.0010 (11)	0.0045 (10)
C16'	0.0492 (15)	0.0344 (12)	0.0406 (12)	−0.0055 (11)	−0.0030 (10)	0.0074 (10)
C17'	0.0413 (13)	0.0309 (11)	0.0402 (12)	−0.0052 (10)	−0.0010 (10)	0.0017 (9)
C18'	0.0409 (13)	0.0298 (12)	0.0456 (13)	−0.0046 (10)	−0.0033 (10)	0.0024 (10)

### Geometric parameters (Å, º)

**Table:**

O1—C1	1.341 (3)	O1'—C1'	1.331 (3)
O1—C3	1.482 (3)	O1'—C3'	1.483 (3)
O2—C1	1.227 (3)	O2'—C1'	1.232 (3)
O3—C7	1.460 (3)	O3'—C7'	1.456 (3)
O3—H3A	0.818 (10)	O3'—H3'A	0.821 (10)
O4—C14	1.372 (3)	O4'—C14'	1.370 (3)
O4—H4A	0.826 (10)	O4'—H4'A	0.821 (10)
O5—C16	1.359 (3)	O5'—C16'	1.365 (3)
O5—H5A	0.8200	O5'—H5'A	0.8200
C1—C17	1.489 (3)	C1'—C17'	1.476 (3)
C2—C3	1.503 (4)	C2'—C3'	1.504 (4)
C2—H2A	0.9600	C2'—H2'A	0.9600
C2—H2B	0.9600	C2'—H2'B	0.9600
C2—H2C	0.9600	C2'—H2'C	0.9600
C3—C4	1.517 (4)	C3'—C4'	1.523 (4)
C3—H3B	0.9800	C3'—H3'B	0.9800
C4—C5	1.507 (4)	C4'—C5'	1.527 (4)
C4—H4B	0.9700	C4'—H4'B	0.9700
C4—H4C	0.9700	C4'—H4'C	0.9700
C5—C6	1.540 (4)	C5'—C6'	1.538 (4)
C5—H5B	0.9700	C5'—H5'B	0.9700
C5—H5C	0.9700	C5'—H5'C	0.9700
C6—C7	1.527 (4)	C6'—C7'	1.533 (4)
C6—H6A	0.9700	C6'—H6'A	0.9700
C6—H6B	0.9700	C6'—H6'B	0.9700
C7—C8	1.532 (4)	C7'—C8'	1.527 (4)
C7—H7A	0.9800	C7'—H7'A	0.9800
C8—C9	1.523 (5)	C8'—C9'	1.542 (4)
C8—H8A	0.9700	C8'—H8'A	0.9700
C8—H8B	0.9700	C8'—H8'B	0.9700
C9—C10	1.520 (4)	C9'—C10'	1.532 (4)
C9—H9A	0.9700	C9'—H9'A	0.9700
C9—H9B	0.9700	C9'—H9'B	0.9700
C10—C11	1.534 (3)	C10'—C11'	1.528 (4)
C10—H10A	0.9700	C10'—H10C	0.9700
C10—H10B	0.9700	C10'—H10D	0.9700
C11—C12	1.524 (3)	C11'—C12'	1.540 (4)
C11—H11A	0.9700	C11'—H11C	0.9700
C11—H11B	0.9700	C11'—H11D	0.9700
C12—C18	1.531 (3)	C12'—C18'	1.520 (3)
C12—H12A	0.9700	C12'—H12C	0.9700
C12—H12B	0.9700	C12'—H12D	0.9700
C13—C18	1.388 (3)	C13'—C18'	1.381 (3)
C13—C14	1.391 (4)	C13'—C14'	1.392 (4)
C13—H13A	0.9300	C13'—H13B	0.9300
C14—C15	1.385 (4)	C14'—C15'	1.379 (4)
C15—C16	1.394 (3)	C15'—C16'	1.387 (4)
C15—H15A	0.9300	C15'—H15B	0.9300
C16—C17	1.425 (3)	C16'—C17'	1.421 (3)
C17—C18	1.419 (3)	C17'—C18'	1.428 (3)
			
C1—O1—C3	117.26 (18)	C1'—O1'—C3'	116.96 (18)
C7—O3—H3A	113 (3)	C7'—O3'—H3'A	103 (3)
C14—O4—H4A	106 (3)	C14'—O4'—H4'A	104 (4)
C16—O5—H5A	109.5	C16'—O5'—H5'A	109.5
O2—C1—O1	120.9 (2)	O2'—C1'—O1'	121.1 (2)
O2—C1—C17	122.7 (2)	O2'—C1'—C17'	122.5 (2)
O1—C1—C17	116.3 (2)	O1'—C1'—C17'	116.3 (2)
C3—C2—H2A	109.5	C3'—C2'—H2'A	109.5
C3—C2—H2B	109.5	C3'—C2'—H2'B	109.5
H2A—C2—H2B	109.5	H2'A—C2'—H2'B	109.5
C3—C2—H2C	109.5	C3'—C2'—H2'C	109.5
H2A—C2—H2C	109.5	H2'A—C2'—H2'C	109.5
H2B—C2—H2C	109.5	H2'B—C2'—H2'C	109.5
O1—C3—C2	109.9 (2)	O1'—C3'—C2'	109.8 (2)
O1—C3—C4	105.53 (19)	O1'—C3'—C4'	105.24 (19)
C2—C3—C4	113.1 (2)	C2'—C3'—C4'	112.8 (2)
O1—C3—H3B	109.4	O1'—C3'—H3'B	109.6
C2—C3—H3B	109.4	C2'—C3'—H3'B	109.6
C4—C3—H3B	109.4	C4'—C3'—H3'B	109.6
C5—C4—C3	114.9 (2)	C3'—C4'—C5'	114.6 (2)
C5—C4—H4B	108.5	C3'—C4'—H4'B	108.6
C3—C4—H4B	108.5	C5'—C4'—H4'B	108.6
C5—C4—H4C	108.5	C3'—C4'—H4'C	108.6
C3—C4—H4C	108.5	C5'—C4'—H4'C	108.6
H4B—C4—H4C	107.5	H4'B—C4'—H4'C	107.6
C4—C5—C6	114.8 (2)	C4'—C5'—C6'	114.1 (2)
C4—C5—H5B	108.6	C4'—C5'—H5'B	108.7
C6—C5—H5B	108.6	C6'—C5'—H5'B	108.7
C4—C5—H5C	108.6	C4'—C5'—H5'C	108.7
C6—C5—H5C	108.6	C6'—C5'—H5'C	108.7
H5B—C5—H5C	107.5	H5'B—C5'—H5'C	107.6
C7—C6—C5	114.4 (2)	C7'—C6'—C5'	114.9 (2)
C7—C6—H6A	108.7	C7'—C6'—H6'A	108.5
C5—C6—H6A	108.7	C5'—C6'—H6'A	108.5
C7—C6—H6B	108.7	C7'—C6'—H6'B	108.5
C5—C6—H6B	108.7	C5'—C6'—H6'B	108.5
H6A—C6—H6B	107.6	H6'A—C6'—H6'B	107.5
O3—C7—C6	110.1 (2)	O3'—C7'—C8'	109.8 (2)
O3—C7—C8	109.6 (2)	O3'—C7'—C6'	110.6 (2)
C6—C7—C8	114.5 (2)	C8'—C7'—C6'	113.3 (2)
O3—C7—H7A	107.5	O3'—C7'—H7'A	107.6
C6—C7—H7A	107.5	C8'—C7'—H7'A	107.6
C8—C7—H7A	107.5	C6'—C7'—H7'A	107.6
C9—C8—C7	114.4 (2)	C7'—C8'—C9'	113.6 (3)
C9—C8—H8A	108.7	C7'—C8'—H8'A	108.8
C7—C8—H8A	108.7	C9'—C8'—H8'A	108.8
C9—C8—H8B	108.7	C7'—C8'—H8'B	108.8
C7—C8—H8B	108.7	C9'—C8'—H8'B	108.8
H8A—C8—H8B	107.6	H8'A—C8'—H8'B	107.7
C10—C9—C8	114.9 (2)	C10'—C9'—C8'	114.1 (2)
C10—C9—H9A	108.5	C10'—C9'—H9'A	108.7
C8—C9—H9A	108.5	C8'—C9'—H9'A	108.7
C10—C9—H9B	108.5	C10'—C9'—H9'B	108.7
C8—C9—H9B	108.5	C8'—C9'—H9'B	108.7
H9A—C9—H9B	107.5	H9'A—C9'—H9'B	107.6
C9—C10—C11	114.1 (2)	C11'—C10'—C9'	114.4 (3)
C9—C10—H10A	108.7	C11'—C10'—H10C	108.7
C11—C10—H10A	108.7	C9'—C10'—H10C	108.7
C9—C10—H10B	108.7	C11'—C10'—H10D	108.7
C11—C10—H10B	108.7	C9'—C10'—H10D	108.7
H10A—C10—H10B	107.6	H10C—C10'—H10D	107.6
C12—C11—C10	112.7 (2)	C10'—C11'—C12'	113.4 (2)
C12—C11—H11A	109.1	C10'—C11'—H11C	108.9
C10—C11—H11A	109.1	C12'—C11'—H11C	108.9
C12—C11—H11B	109.1	C10'—C11'—H11D	108.9
C10—C11—H11B	109.1	C12'—C11'—H11D	108.9
H11A—C11—H11B	107.8	H11C—C11'—H11D	107.7
C11—C12—C18	112.0 (2)	C18'—C12'—C11'	111.6 (2)
C11—C12—H12A	109.2	C18'—C12'—H12C	109.3
C18—C12—H12A	109.2	C11'—C12'—H12C	109.3
C11—C12—H12B	109.2	C18'—C12'—H12D	109.3
C18—C12—H12B	109.2	C11'—C12'—H12D	109.3
H12A—C12—H12B	107.9	H12C—C12'—H12D	108.0
C18—C13—C14	122.0 (2)	C18'—C13'—C14'	121.6 (2)
C18—C13—H13A	119.0	C18'—C13'—H13B	119.2
C14—C13—H13A	119.0	C14'—C13'—H13B	119.2
O4—C14—C15	122.8 (2)	O4'—C14'—C15'	122.5 (2)
O4—C14—C13	116.8 (2)	O4'—C14'—C13'	116.7 (2)
C15—C14—C13	120.4 (2)	C15'—C14'—C13'	120.8 (2)
C14—C15—C16	119.3 (2)	C14'—C15'—C16'	119.0 (2)
C14—C15—H15A	120.4	C14'—C15'—H15B	120.5
C16—C15—H15A	120.4	C16'—C15'—H15B	120.5
O5—C16—C15	115.8 (2)	O5'—C16'—C15'	115.6 (2)
O5—C16—C17	123.2 (2)	O5'—C16'—C17'	122.9 (2)
C15—C16—C17	121.0 (2)	C15'—C16'—C17'	121.5 (2)
C18—C17—C16	118.8 (2)	C16'—C17'—C18'	118.2 (2)
C18—C17—C1	125.7 (2)	C16'—C17'—C1'	116.0 (2)
C16—C17—C1	115.6 (2)	C18'—C17'—C1'	125.8 (2)
C13—C18—C17	118.6 (2)	C13'—C18'—C17'	118.8 (2)
C13—C18—C12	116.4 (2)	C13'—C18'—C12'	116.2 (2)
C17—C18—C12	125.0 (2)	C17'—C18'—C12'	124.9 (2)

### Hydrogen-bond geometry (Å, º)

**Table:**

*D*—H···*A*	*D*—H	H···*A*	*D*···*A*	*D*—H···*A*
O5—H5*A*···O2	0.82	1.83	2.549 (3)	146
O5′—H5′*A*···O2′	0.82	1.82	2.540 (3)	146
O4—H4*A*···O3^i^	0.83 (2)	1.93 (3)	2.745 (3)	171 (3)
O4′—H4′*A*···O3′^ii^	0.82 (3)	1.94 (3)	2.740 (3)	163 (4)
O3—H3*A*···O4^iii^	0.82 (3)	2.29 (3)	3.080 (3)	162 (3)
O3′—H3′*A*···O4′^iii^	0.82 (3)	2.27 (3)	3.067 (3)	165 (4)

Symmetry codes: (i) *x*+1, *y*+1, *z*; (ii) *x*−1, *y*+1, *z*; (iii) *x*, *y*−1, *z*.
